# Contralateral artery enlargement predicts carotid plaque progression based on machine learning algorithm models in apoE^−/−^ mice

**DOI:** 10.1186/s12938-016-0265-z

**Published:** 2016-12-28

**Authors:** Bing Li, Yun Jiao, Cong Fu, Bo Xie, Genshan Ma, Gaojun Teng, Yuyu Yao

**Affiliations:** 1 Department of Cardiology, Zhongda Hospital, Medical School of Southeast University, 87 Dingjiaqiao, Nanjing, 210009 Jiangsu China; 2 Jiangsu Key Lab of Molecular and Function Imaging, Department of Radiology, Zhongda Hospital, Medical School of Southeast University, Nanjing, 210009 China

**Keywords:** Low shear stress, Atherosclerosis, Machine learning algorithms, Animal model

## Abstract

**Background:**

This study specifically focused on anatomical MRI characterization of the low shear stress-induced atherosclerotic plaque in mice. We used machine learning algorithms to analyze multiple correlation factors of plaque to generate predictive models and to find the predictive factor for vulnerable plaque.

**Methods:**

Branches of the left carotid artery in apoE^−/−^ and C57BL/6J mice were ligated to produce the partial left carotid artery model. Before surgery, and 7, 14, and 28 days after surgery, in vivo serial MRI measurements of carotid artery diameter were obtained. Meanwhile, proximal blood flow was evaluated. After image acquisition and animal sacrifice, carotid arteries were harvested for histological analysis. Support vector machine (SVM) and decision tree (DT) were used to select features and generate predictive models of vulnerable plaque progression.

**Result:**

Seven days after surgery, neointima formation was visualized on micro-MRI in both apoE^−/−^ and C57BL/6J mice. Ultrasonography showed that blood flow had significantly decreased compared to that in the contralateral artery. Partial ligation of the carotid artery for 4 weeks in apoE^−/−^ mice induced vulnerable plaque; however, in C57BL/6J mice this same technique performed for 4 weeks induced arterial stenosis. Contralateral carotid artery diameter at 7 days after surgery was the most reliable predictive factor in plaque progression. We achieved over 87.5% accuracy, 80% sensitivity, and 95% specificity with SVM. The accuracy, sensitivity, and specificity for the DT classifier were 90, 90, and 90%, respectively.

**Conclusions:**

This study is the first to demonstrate that SVM and DT methods could be suitable models for identifying vulnerable plaque progression in mice. And contralateral artery enlargement can predict the vulnerable plaque in carotid artery at the very early stage. It may be a valuable tool which helps to optimize the clinical work flow process by providing more decision in selecting patients for treatment.

## Background

Atherosclerosis, a complex multifactorial disease, is the leading cause of death worldwide. It is characterized as patchy intimal plaques that encroach on the lumen of medium-sized and large arteries; the plaques comprise lipids, inflammatory cells, smooth muscle cells, and collagen matrix [1]. The etiology of atherosclerosis is complex, poorly understood, and remains controversial [2]. Extensive evidence indicates that blood flow-induced shear stress plays a critical role in atherogenesis in humans and animals [3, 4]. Shear stress is biomechanical force acting on the endothelium, which is determined by blood flow, vessel geometry, and fluid viscosity [5]. Researchers have found that low and oscillatory endothelial shear stress-induced atherosclerotic lesions form at specific arterial regions [6].

Two common animal models are used to mimic the low shear stress that, in humans, contributes to carotid atherosclerosis. One is carotid constriction, using a constrictive cuff [7] or a conical cast [8]. The other is ligation of the left external and internal carotid arterial branches [9]. Merino et al. [10] used well-examined apoE^−/−^ mice to generate atherosclerosis by ligating the carotid artery, inducing low blood flow velocity, and generating shear stress, which is evidenced by plaque formation in the carotid artery, infiltration of monocytes and macrophages in the plaque, increase in pro-inflammatory cytokines, and decrease in blood velocity. This model shows that this could be a potential animal model to study human cardiovascular disease.

Generally, animal models have the advantage of a shorter time to lesion development and allow the investigation of pathology in a large number of individuals [11]. Traditionally, animal research has resulted in great benefits for humans, but it requires that researchers keep sacrificing animals to gather the beneficial information. This situation changed with the advent of molecular imaging techniques and bioinformation. High-resolution magnetic resonance imaging (MRI) [12], ultrasound [13], and positron emission tomography (PET) [14] are noninvasive and permit longitudinal studies of plaque initiation, growth, and regression in the same animal. Micro-MRI plays a significant role in the study of experimental atherosclerosis. MRI can effectively assess luminal narrowing, plaque size, and morphology [15]. Despite these advantages, MRI still suffers from low spatial resolution and motion artifacts in the artery, and thus remains limited in its ability to identify the vulnerable components of plaque [16]. The features seen on the MRI images are not exactly the same with those found in the histological examination. In order to improve the accuracy of MRI in the diagnosis of plaque, there is a need for extracting discriminate features on the MRI images [17, 18].

The support vector machine (SVM) is one of the most popular machine learning algorithms, originally introduced by Vapnik [19]. SVM was successively implemented for the classification of mice with common carotid artery abnormalities [20] and cancer tissue samples [21]. The goal of SVM is to produce an algorithmic model that predicts the target values of the test data given only the test data attributes. Decision tree (DT) learning uses a decision tree as a predictive model that maps observations about an item to conclusions about the item’s target value. It is one of the predictive modeling approaches used in statistics, data mining, and machine learning. It has very simple procedures to conduct classifications, which make it very fast [22].

In this study, we focused on establishing a predictive model to evaluate vulnerable atherosclerotic plaque formation and progress. We established low shear stress model of atherosclerosis in apoE^−/−^ mice and C57BL/6J mice. MRI was used to collect the data of plaque formation and progress. The plaques in the C57BL/6J group were “stable” and the plaques in the apoE^−/−^ group were “vulnerable” according the ultima pathology detection and vulnerability index formula. And we used two machine learning algorithms to analyze the multiple correlation factors of low shear stress induced plaque in apoE^−/−^ and C57BL/6J mice, and to try to find the predictive factor for vulnerable plaque progression. Analysis shows that the proposed feature is clinically significant.

## Methods

### Animal model

All procedures in the present study were conducted in accordance with the National Instituted of Health Guide for the Care and Use of Laboratory Animals and approved by the Care of Experimental Animals Committee of the Southeast University (Approval ID: SYXK-2012.3921). Male apoE^−/−^ mice (n = 20), 9 weeks old, and a control group of male C57BL/6J mice (n = 20), 9 weeks old, were obtained from the Changzhou Cavens Laboratory Animal Ltd. (Changzhou, China, approval ID:SCXK2011-0003). The animals received a western-type diet (high levels of fat and cholesterol added to grain-based chow diets) for 2 weeks prior to surgery. After all the mice had eaten this diet for 2 weeks, their left external and internal carotid arterial branches were isolated and ligated with 6-0 silk suture. The contralateral right carotid artery was sham-operated to serve as an intra-animal control.

### In vivo serial MRI studies

After the surgery, all mice underwent MRI examinations to measure the diameter of the lumen wall at 7, 14, and 28 days. All scanning was performed on a Micro-MRI animal scanner (7.0T Bruker PharmaScans, Bruker Biospin, Ettlingen, Germany). Isoflurane anesthesia (2%) in each mouse was induced and maintained in medical-grade air and monitored using small animal instrument monitoring (reduced respiratory rate to 40 breath/min) to eliminate the influence of respiratory movement on MRI image quality. All animals were placed in the supine position. Eighteen contiguous, 500-μm thick axial slices spanning from the neck region of the mouse were acquired using a spin echo sequence. MRI images were obtained using black-blood T2 to proton density (PD)-weighted multi-spin multi-echo (MSME) sequence and a dedicated mouse volume coil. Imaging parameters were as follows: TR 1206.9 ms, TE 12.8/34.2 ms, FOV 2.5 cm, FA was 180°, matrix size 256 × 256, and in-plane resolution 141 × 141 × 800 μm^3^; slice thickness of 0.5 mm, and four excitations. Fat suppression was performed for proton density and T2-weighted imaging. The total MR scanning procedure required approximately 50 min [23].

The obtained images were analyzed using ImageJ 1.41 software (ImageJ, NIH, Bethesda, MD, USA). Continuous imaging on slices allowed us to measure the diameters and areas of vascular intima and adventitia by computer-assisted planimetry. Also we calculate the volume of plaque through the plaque area of each slice multiplied by the thickness of each slice.

### Ultrasonographic imaging and pulse-wave Doppler measurement

The blood flow velocity was measured in both carotids using an ultrasonic instrument (Vevo 2100, VisualSonices Inc, Toronto, Canada) at 7, 14, and 28 days after the surgery. Each mouse was anesthetized and maintained under isoflurane anesthesia (2%) and laid on a platform in the supine position with all legs taped to ECG electrodes for heart rate monitoring. An MS 400 (30 MHz centerline frequency) probe was used to measure the frequency of the common carotid artery blood flow to the midpoint spectrum and to measure the blood flow velocity, as previously described [24]. Shear stress was calculated using the following formula: SS = 4 μVm/Ds [25], where μ is the blood viscosity (taken as 0.035 poise), Vm is the mean flow velocity (mm/s), and Ds is the lumen diameter of the targeted carotid artery (mm).

### Serum lipid measurement

After MRI and ultrasound examination at 28 days, blood samples were taken while the mice were still anesthetized by angular vein puncture at the time of sacrifice. Serum was separated and serum total cholesterol (TC) and low-density lipoprotein (LDL) were determined by using an automatic biochemistry analyzer.

### Histological examination

At the end of the procedure, carotid arteries were collected from sacrificed mice, immediately embedding OCT compound, and rapidly frozen using liquid nitrogen. Serial cryosections of 8 μm were cut on a Lecia CM1950 Cryostat (Lecia Instruments, Heidelberg, Germany). The serial cryosections were stained with hematoxylin-eosin, Oil red O, Masson’s trichrome, respectively. For immunohistochemical analyses, α-smooth muscle actin (α-SMC, Abcam, Cambridge, UK) and MAC-3 (BD Biosciences Pharmingen, San Diego, CA) stains were used for detection of smooth muscle cells and macrophages, respectively. ImageJ 1.41 software was used to measure the content of collagen, lipid, macrophages and vascular smooth muscle cells. The vulnerability index was calculated using the following formula:positive staining area of (macrophages + lipid)/positive staining area of (SMCs + collagen) [26].

### Machine learning algorithms

We selected SVM because it has been shown empirically to achieve good generalization performance on a wide variety of classification problems. The attribute weight, *w*, describes the separating hyper plane of a liner SVM [27]. Parameter *w* of SVM indicated the contribution of the feature to SVM. In this report, the absolute value for each weight means the importance for prediction of carotid vulnerable plaque progress. Positive w means increased in the normal control (decreased in apoE^−/−^ group), while negative value means decreased activities in the normal control (increased in apoE^−/−^ group).

We selected decision tree because it uses a white box model, which is simple to understand and interpret. A decision tree is a flowchart-like structure in which the internal node represents a “test” on an attribute, each branch represents the outcome of the test, and each leaf node represents a class label (decision made after computing all attributes). The paths from root to leaf represent classification rules [28].

We selected ten features as follows: the lumen diameter of left carotid artery (LCA) and right carotid artery (RCA), the lumen area of LCA and RCA, the blood flow velocity of LCA and RCA, the ratio of the lumen diameter of LCA to RCA, the ratio of the lumen area of LCA to RCA, the ratio of the blood flow velocity of LCA to RCA and the plaque volume of LCA at 7 days separately.

### Predictive model evaluation

SVM and DT were applied by use of WEKA (Version 3.6, The University of Waikato, Hamilton, New Zealand). We evaluated the predictive models generated by these two machine-learning algorithms based on tenfold cross-validation; we randomly divided the data into ten groups of four subjects each (i.e., one-tenth of subjects were placed in each group), with controls being mixed with vulnerable plaque subjects. We ran ten iterations, using 90% of subjects for classifier generation (i.e., training) and the remaining 10% for testing. After cycling through all ten partitions for classification, every group was used for testing, and each group appeared in a training set every time except when it was used for testing [29].

We evaluated the performance of each predictive model based on the following metrics: true-positive rate, false-positive rate, accuracy, and area under the receiver operating characteristic (ROC) curve (AUC). Let N_TP_ denote the number of mice with vulnerable plaque (28-day pathology confirmed as vulnerable plaque) correctly predicted as having vulnerable plaque, let N_FP_ denote the number of control subjects incorrectly identified as having vulnerable plaque, let N_TN_ denote the number of control subjects correctly identified, and let N_FN_ denote mice with vulnerable plaque incorrectly identified as control subjects. Accuracy (ACC) is the proportion of correctly labeled instances in the study, defined as ACC = (N_TP_ + N_TN_)/(N_TP_ + N_TN_ + N_FP_ + N_FN_). The true-positive rate (TPR), or sensitivity, is the proportion of positive instances that were correctly reported as being positive (e.g., TPR = N_TP_/[N_TP_ + N_FN_]). The false-positive rate (FPR), or (1 − specificity), is the proportion of negative instances that were erroneously reported as being positive (FPR = N_FP_/[N_FP_ + N_TN_]). For a diagnostic system with probabilistic output, the ROC curve is a graphical plot of TPR (y-axis) against FPR (x-axis), because the discrimination threshold is varied. We used AUC as an additional measure of the performance for each model.

### Statistics

Data were evaluated by use of SPSS 19 (SPSS, Chicago, IL, USA). Differences for two groups were compared by t test. Differences were considered statistically significant at a value of P < 0.05. Data are expressed as mean ± standard deviation of the mean (SD).

## Results

### In vivo MRI of the vessel wall morphology

**Fig. 1:**
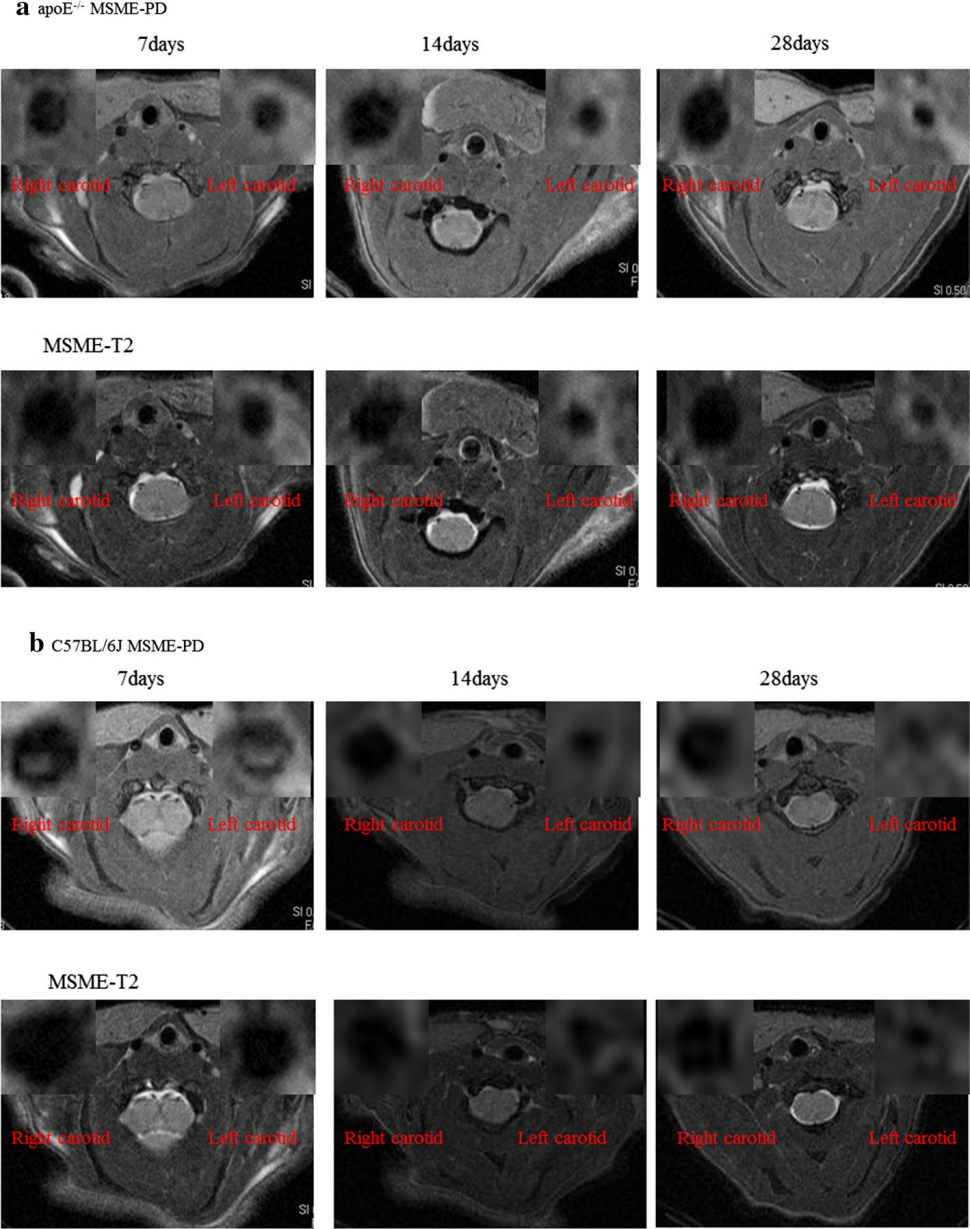
MR images of cross-sectional view through the carotid depicting progression of atherosclerotic plaque development over 4 weeks period in representative animals from apoE^−/−^ (**a**) and C57BL/6J (**b**) groups. **a** Example of MR images from days 7 to 28 after the surgery of the apoE^−/−^ group. The images note the pronounced thickening of the vessel wall in the left carotid and the slight expansion of the vessel in the right carotid over time. **b** Example of MR images from days 7 to 28 after the surgery of the C57BL/6J group. The images show a significant stenosis of the left carotid artery and a slight expansion of the right carotid artery

**Fig. 2:**
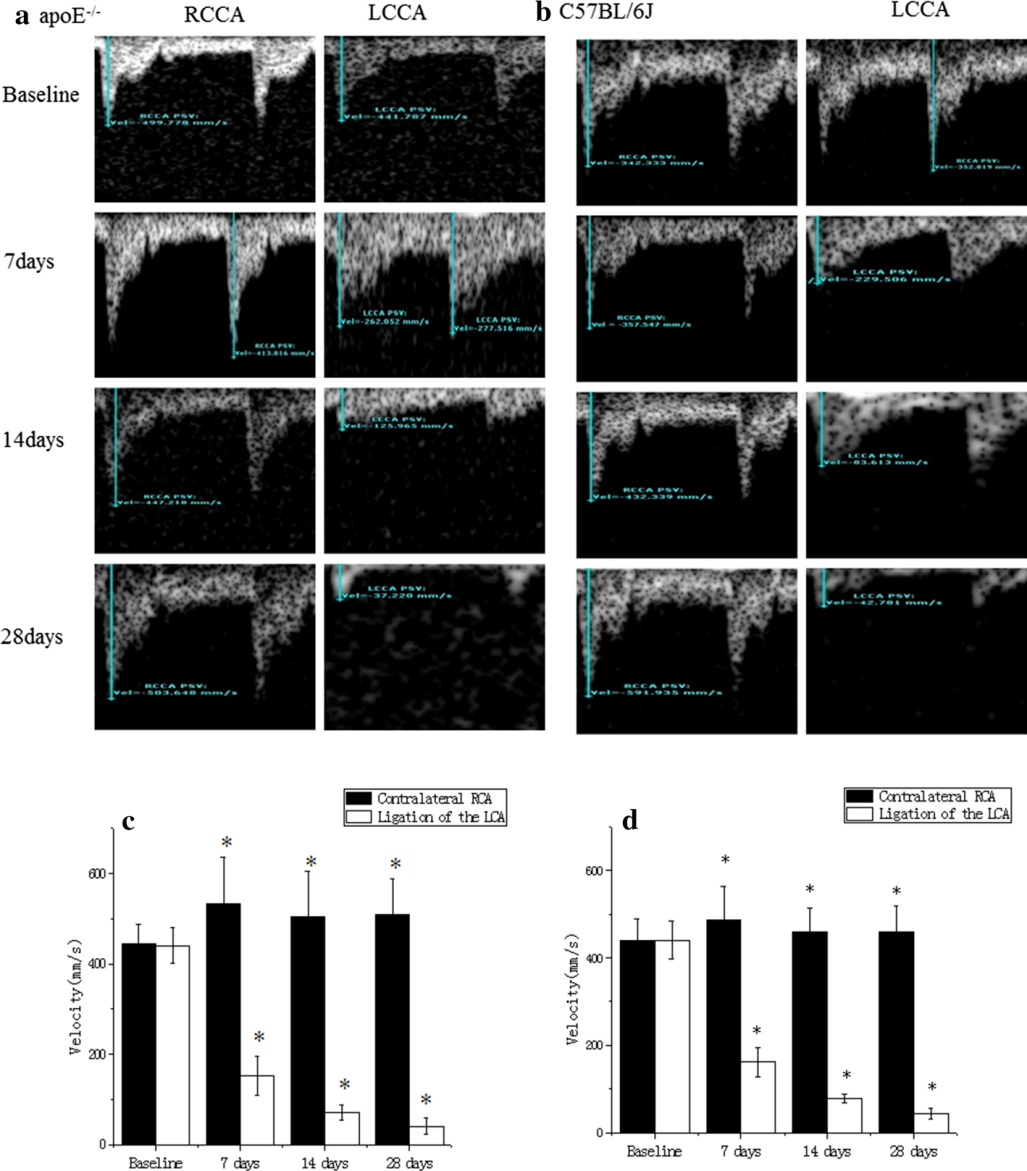
Ultrasound recordings of the carotid flow velocities in apoE^−/−^ (**a**) and C57BL/6J (**b**) groups. A1: baseline flow velocity in apoE^−/−^ group. A2: flow velocity in the carotid 7 days after carotid surgery in apoE^−/−^ group. A3: flow velocity in the carotid 14 days after carotid surgery in apoE^−/−^ group. A4: flow velocity in the carotid 28 days after carotid surgery in apoE^−/−^ group. B1: baseline flow velocity in C57BL/6J group. B2: flow velocity in the carotid 7 days after carotid surgery in C57BL/6J group. B3: flow velocity in the carotid 14 days after carotid surgery in C57BL/6J group. B4: flow velocity in the carotid 28 days after carotid surgery in C57BL/6J group. The blood flow velocity of apoE^−/−^ (**c**) and C57BL/6J (**d**) groups were calculated from pre-surgery tracings and 7, 14 and 28 days after surgery. Data are mean ± SD. There was a highly significant of velocity at baseline. *P < 0.05, n = 40, compared to baseline

MRI provided clear cross-sectional images of atherosclerotic lesions in the carotid artery. Before surgery, both sides of the common carotid artery wall on T2WI, PDWI was homogeneously, slightly high signal (compared with muscle tissue around the signal). From days 7 to 28, there was a gradual arterial stenosis in the lumen of the left common carotid, and the lumen diameter of the contralateral common carotid progressively increased in the apoE^−/−^ group. There is a significant reduction in the diameter of the left common carotid artery 0.42 ± 0.01 mm at 7 days, 0.29 ± 0.02 mm at 14 days, 0.23 ± 0.01 mm at 28 days, compared to the diameter of the left carotid artery before surgery (0.55 ± 0.02 mm, Fig. 1). We also observed a significant expansion in the diameter of the right common carotid artery 0.64 ± 0.02 mm at 7 days, 0.66 ± 0.03 mm at 14 days and 0.67 ± 0.01 mm at 28 days compared to the diameter of the right carotid artery 0.56 ± 0.01 mm before surgery. In the C57BL/6J mice group, ligation also induced arterial stenosis. But we observed a mild reduction in the diameter of the left common carotid artery 0.44 ± 0.02 mm at 7 days and significantly reduced diameter of the left carotid artery 0.30 ± 0.03 mm at 14 days and 0.27 ± 0.01 mm at 28 days, compared to the diameter of the left carotid artery at baseline (0.55 ± 0.02 mm, Fig. 2). We observed a slight expansion in the diameter of the right common carotid artery 0.61 ± 0.01 mm at 7 days, 0.62 ± 0.02 mm at 14 days, and 0.63 ± 0.01 mm at 28 days compared to the diameter of the right carotid artery 0.55 ± 0.03 mm at baseline. We also calculated the plaque volume using MRI data, as shown in Table 1. A significantly larger plaque volume is found in apoE^−/−^ compared to C57BL/6J mice (P < 0.05).

**Table 1:** Mean and standard deviation for monitoring variables of apoE^−/−^ and C57BL/6J mice group at 7 days

	Predictive power	ApoE^−/−^	C57BL/6J	P value
Lumen diameter of LCA (mm)	−0.21	0.42 ± 0.01	0.44 ± 0.02	0.015
Lumen diameter of RCA (mm)	−1.48	0.65 ± 0.02	0.61 ± 0.01	<0.001*
Lumen area of LCA (mm^2^)	0.09	0.14 ± 0.01	0.15 ± 0.02	0.030
Lumen area of RCA (mm^2^)	−1.31	0.34 ± 0.02	0.30 ± 0.02	<0.001**
Blood flow velocity of LCA (mm/s)	0.47	152.42 ± 43.67	162.17 ± 33.41	0.438
Blood flow velocity of RCA (mm/s)	0.24	532.84 ± 104.35	487.82 ± 76.34	0.128
The ratio of the lumen diameter of LCA to RCA	0.63	0.64 ± 0.03	0.72 ± 0.05	<0.001***
The ratio of the lumen area of LCA to RCA	0.72	0.41 ± 0.05	0.53 ± 0.06	<0.001****
The ratio of the blood flow velocity of LCA to RCA	1.09	0.29 ± 0.08	0.34 ± 0.09	1.011
Plaque volume of LCA (mm^3^)	0.56	0.77 ± 0.14	0.53 ± 0.15	0.001

Data are presented as mean ± SD. Predictive power is the parameter w of SVM indicated the contribution of the feature to SVM. It means the importance for prediction of carotid vulnerable plaque progress. Positive means increased in the normal control (decreased in apoE^−/−^ group), while negative is contrast. P value indicated the result of t tests between two groups. * P = 1.29 × 10^−7^, ** P = 3.38 × 10^−7^, *** P = 2.38 × 10^−6^, **** P = 1.24 × 10^−6^

### Blood velocity and shear stress

In both apoE^−/−^ and C57BL/6J mice, the blood flow velocity in the surgery side decreased significantly compared with the contralateral side as showed in Table 2. And according to the formula SS = 4 μVm/Ds, the shear stress in the surgery side also decreased significantly compared with that in the contralateral side. As time went on, the blood flow velocity and shear stress declined gradually, and no statistically significant difference between apoE^−/−^ and control mice was found during the study.

**Table 2:** Changes of shear stress in low velocity regions between two groups detected by MRI and US measurement before and after surgery

	ApoE^−/−^				C57BL/6J			
	7 days		14 days		7 days		14 days	
	LCA	RCA	LCA	RCA	LCA	RCA	LCA	RCA
Vm (mm/s)	152.4 ± 43.7	532.8 ± 104.4	71.3 ± 16.6	505.3 ± 98.7	162.2 ± 33.4	487.8 ± 76.3	79.1 ± 9.9	458.9 ± 55.8
Dr (mm)	0.42 ± 0.01	0.64 ± 0.02	0.29 ± 0.02	0.66 ± 0.03	0.44 ± 0.02	0.61 ± 0.01	0.3 ± 0.03	0.62 ± 0.02
SS (dyn/cm^2^)	50.4 ± 14.6*	113.1 ± 22.1	34.3 ± 6.0*^,#^	109.8 ± 21.9	51.3 ± 11.2*	111.1 ± 17.4	37.2 ± 5.8*^,#^	103.6 ± 13.1

Data are mean ± SD

*Vm* mean velocity of carotid artery, *Ds* end-systolic diameters of carotid artery, *SS* shear stress of carotid artery, *μ* blood viscosity of mice, *SS* 4 μVm/Ds

* P < 0.05, comparing the shear stress of LCA with the shear stress of RCA. ^# ^P < 0.05, comparing the shear stress at 14 days with the shear stress at 7 days

### Serum lipid levels

During the experiment, there was no significant difference in body weight between apoE^−/−^ (28 ± 1.28 g) and C57BL/6J mice (27.6 ± 1.46 g, P > 0.05). The serum levels of total cholesterol and LDL in the apoE^−/−^ group (13.37 ± 6.24 and 3.98 ± 1.24 mmol/L) were significantly higher compared with that in the C57BL/6J group (2.96 ± 0.28 and 1.42 ± 0.49 mmol/L, P < 0.05).

### Morphometric analyses

The composition of plaque including macrophages, SMCs, collagen, and lipids was measured by histological and immunohistochemical staining (Table 3).

**Table 3:** Comparison of plaque morphological parameters after 28 days

	ApoE^−/−^	C57BL/6J
Macrophages (%)	29.4 ± 8.7	18.9 ± 6.5*
SMCs (%)	6.8 ± 2.3	26.2 ± 9.8*
Lipids (%)	42 ± 10.6	10.9 ± 2.4*
Collagen (%)	15.8 ± 3.5	44.6 ± 13.7*
The vulnerability index	2.81 ± 0.21	0.42 ± 0.13*

Data are presented as mean ± SD

* P < 0.05, comparing the C57BL/6J group with the apoE^−/−^ group

**Fig. 3:**
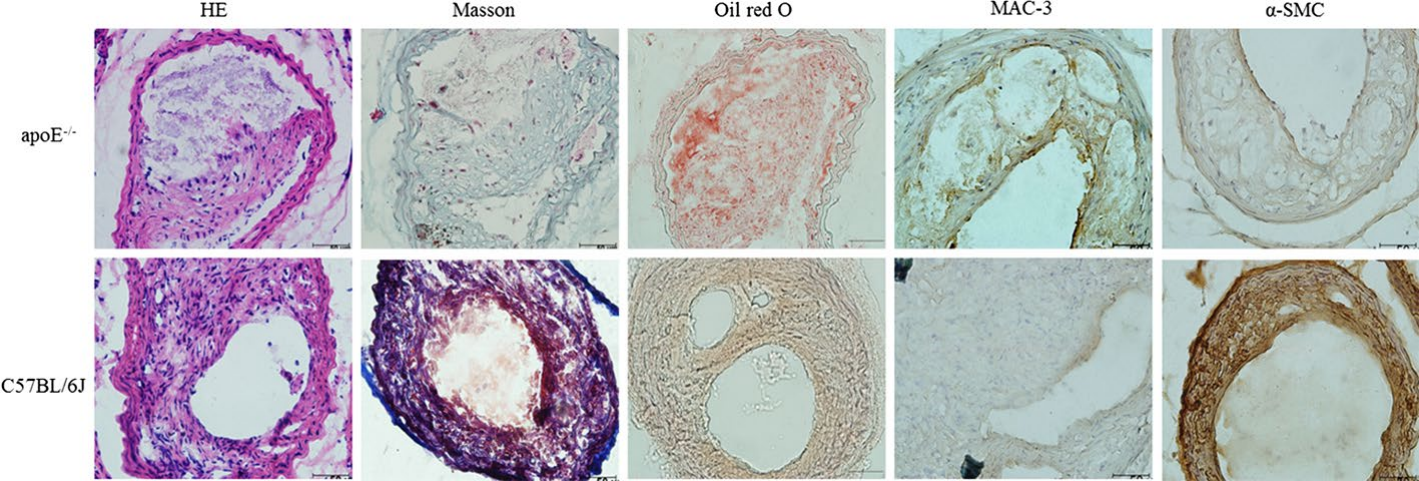
Histopathological and immunohistochemical staining showing plaque composition in apoE^−/−^ and C57BL/6J groups after 28 days. Cross-sections of carotid arteries in different groups were stained for HE, collagen (Masson’s trichrome) and lipids (Oil red O), macrophages (MAC-3) and vascular SMCs (α-actin). *Scale bar* 50 μm

The plaque in the apoE^−/−^ group had a higher content of lipids and macrophages and a lower content of collagen than the C57BL/6J group (Fig. 3). The stenosis in C57BL/6J mice at 4 weeks contained smooth muscle cells. The vulnerability index of the apoE^−/−^ group (2.81 ± 0.21) was higher than that of the C57BL/6J group (0.42 ± 0.13, P < 0.05). According to the diagnostic criteria of vulnerable plaque established by Naghavi et al. [30], the apoE^−/−^ group had a higher accumulation of lipids and macrophages, a lower content of collagen, thinner fibrous caps, and an increased vulnerability index. So we recognized that all the plaque in apoE^−/−^ mice was vulnerable; however, the plaque in C57BL/6J mice was stable, because the plaques contained lots of smooth muscle cells.

### Results of the predictive models

The performance of the SVM-based model was good with a sensitivity of 95%, specificity of 80%, and accuracy of 87.5% (Table 4). The predictive value (weight) of each feature was estimated in predicting the final result by SVM (Table 1). We found that lumen diameter of RCA at 7 days was the most important feature in the SVM-based model with the highest absolute value (*w* = −1.48).

**Table 4:** The statistical features of SVM and DT

Model	Accuracy (%)	Sensitivity (%)	Specificity (%)	Area under ROC (AUC)
SVM	87.5	80	95	0.875
DT	90	90	90	0.881

**Fig. 4:**
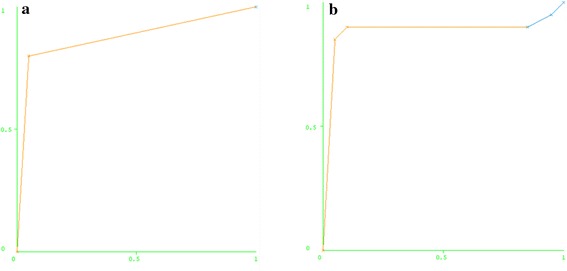
**a** ROC curve for predictions using SVM. **b** ROC curve for predictions using DT

The DT only selected the lumen diameter of RCA at 7 days with sensitivity of 90%, specificity of 90% and accuracy of 90% (Table 4). Figure 4 shows the ROC for predictions using SVM and DT.

**Fig. 5:**
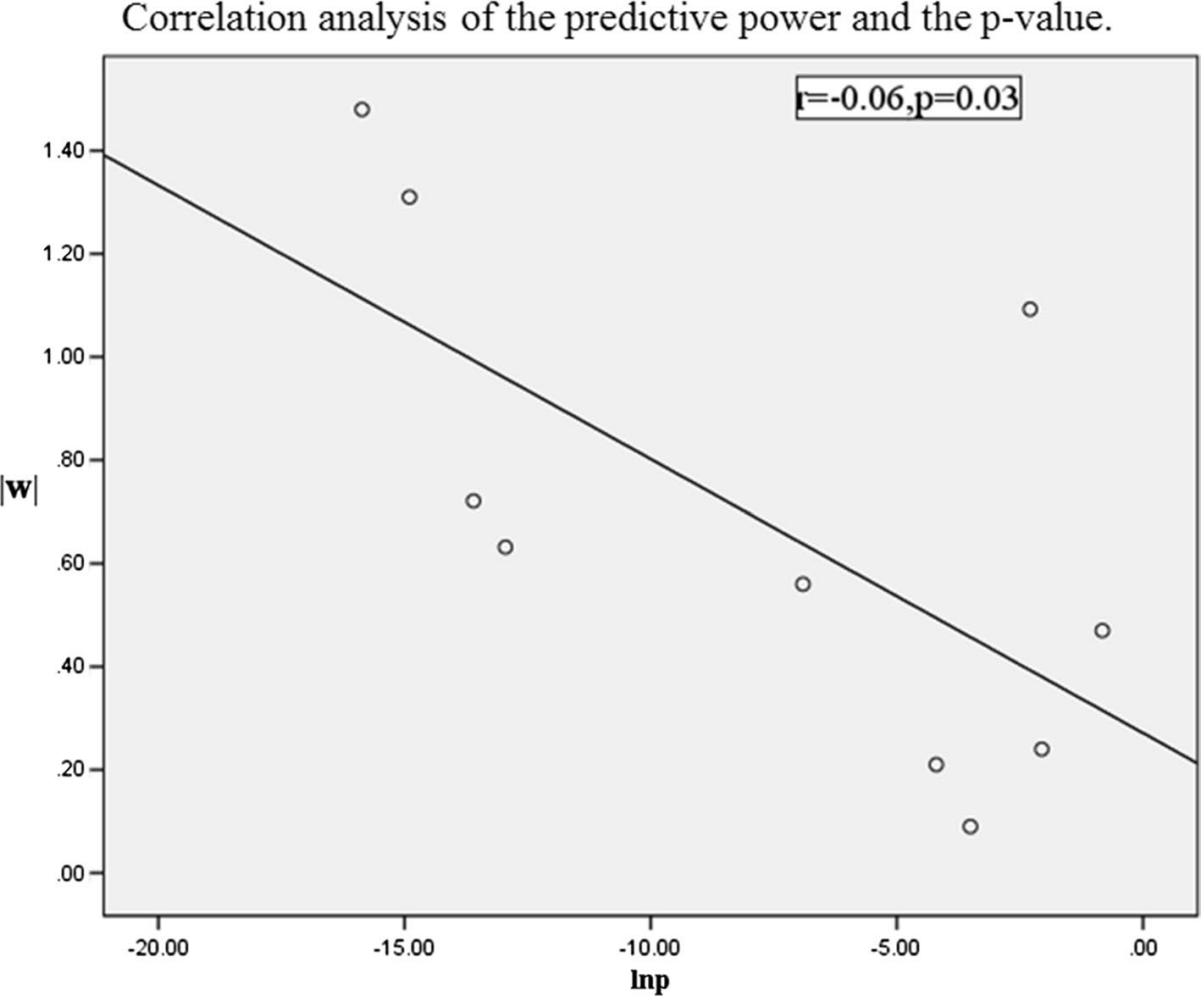
Correlation analysis of the predictive power and the P value. |*w*| represents the absolute value of predictive power calculated by SVM. lnP represents the natural logarithm of P values calculated by t test

We also applied two sample t tests between groups to find statistically significant parameters. The significant features, their respective range (mean ± standard deviation) for both classes, and the P values were shown in the Table 1. It was evident that four features have a P value less than 0.01, including the lumen diameter of RCA at 7 days, these features can be considered significant enough for classification. The lumen diameter of RCA at 7 days was both selected by the machine learning method and the traditional statistical methods as one of the significant features. Because some P values are too small, we chose a natural logarithm of P values. The absolute value of predictive power and P value using the natural logarithm determined that every feature was associated through statistical correlation analysis (r = −0.66, P = 0.03, Fig. 5). It showed that our predictive method was correlated with the traditional method.

## Discussion

We applied the feature selection method based on serial MRI and ultrasound data to search the optimized discriminative predictive factor for vulnerable plaque progression in the early stage of atherosclerosis. The SVM classifier and DT classifier were implemented for the classification of mice with plaque progression. Our study first demonstrates that contralateral artery enlargement can predict carotid plaque progress in apoE^−/−^ mice in the very early stage.

For centuries, researchers have made much progress in understanding the anatomic initiation and progression of atherosclerosis. But how to identify patients at high risk for an acute cardiovascular event and assessing the total atherosclerotic burden are clinically important. New methods have constantly emerged for assessment of atherosclerotic plaque vulnerability, such as computational modeling, molecular imaging, and mechanical analysis [31–33]. However, methods to identify the best predictors and to predict plaque rupture are currently lacking [22].

In this study, we ligated the left external and internal carotid arterial branches to cause plaque in atherosclerosis-prone apoE^−/−^ mice and athero-resistant C57BL/6J mice. In apoE^−/−^ mice, this process bears much resemblance to spontaneous atherosclerosis in terms of both etiology and pathogenesis. From 1 to 4 weeks, the left carotid artery displayed progressive stenosis based on Micro-MRI. At 4 weeks, the results of the left carotid artery’s pathologic examination showed a higher content of lipids and macrophages and a lower content of collagen, indicating that the plaque was vulnerable. But in C57BL/6J mice, although the left carotid artery also displayed progressive stenosis on Micro-MRI, the immunohistochemical staining showed the stenosis in C57BL/6J mice at 4 weeks contained a number of smooth muscle cells. We therefore presumed that the plaque was stable. This is consistent with previous reports that indicate that ligation of C57BL/6J left external and internal carotid branches causes vascular remodeling in a mouse model [9]. We used ultrasound to measure flow velocity from 1 to 4 weeks and found that partial ligation induced low blood velocity. Both apoE^−/−^ and C57BL/6J mice showed significantly decreased flow velocity in the left carotid artery, but in the contralateral artery the flow velocity is increased. Using values of flow velocity and vessel dimensions, we calculated the shear stress. Low shear stress in the left carotid artery caused vulnerable plaque in apoE^−/−^ mice, but caused stable plaque in C57BL/6J mice. In accord with previous reports, plasma cholesterol levels and LDL lipoprotein in apoE^−/−^ mice were significantly higher than that in C57BL/6J mice. In addition, histology results confirmed the different plaque composition between two strains. Hoving et al. [34] showed carotid arteries of atherosclerosis-prone apoE^−/−^ mice and C57BL/6J mice respond very differently to irradiation. This might be partly explained by higher base-line expressions of inflammatory VCAM-1 and thrombotic mediator thrombomodulin. Low shear stress, high plasma cholesterol levels and different gene expression may be involved in the initiation of partial ligation-induced vulnerable atherosclerotic plaque formation in apoE^−/−^ mice. As a matter of fact, the plaque volume of apoE^−/−^ mice is larger than that in C57BL/6J mice.

In this study, we focused on establishing a predictive model to evaluate vulnerable atherosclerotic plaque formation and progress. Using MRI, we serially detected left and right carotid artery lumen diameter, lumen area, and plaque volume from 7 days to 4 weeks. The SVM is widely used in the prediction model [35, 36]. Our results demonstrate that the lumen diameter of RCA at 7 days was the most important feature by SVM. DT has its own feature selection, which is based on the result of SVM, and is performed within the DT training step. The lumen diameter of RCA at 7 days was selected as the only feature by DT in predicting vulnerable plaque and proved to be efficient. The regular statistical method gave the significance of the features, but it could not verify whether the significance feature could reflect the results of plaque vulnerability. Our models not only provided the significance of the features but also selected one of the most important features. The reliability of our model is proven by its accuracy, sensitivity, and specificity. To the best of our knowledge, this is the first study to use SVM and DT as a predictive model to predict plaque progression in an animal model at the early stage of the disease. Plaque identification from images is very difficult; plaque characterization has been carried out by different researchers and physicians. It is difficult to accurately capture and differentiate plaque objectively. With the current model, our system can predict two types of plaque formation with a high accuracy, sensitivity, and specificity. We used the diameter of the right artery of apoE^−/−^ mice at 7 days to accurately predict the vulnerable plaque in the left carotid artery. This method is fairly objective and simple; the researcher can easily measure the diameter with Micro-MRI.

## Conclusion

This study demonstrates that with MRI data, the SVM and DT methods could be suitable models for identifying vulnerable plaque progression in mice. Contralateral artery enlargement can predict carotid plaque progression in apoE^−/−^ mice in the very early stage.
